# Barriers and Facilitators in Implementing Non-Face-to-Face Chronic Care Management in an Elderly Population with Diabetes: A Qualitative Study of Physician and Health System Perspectives

**DOI:** 10.3390/jcm7110451

**Published:** 2018-11-20

**Authors:** Alessandra N. Bazzano, M. Kristina Wharton, Alisha Monnette, Elizabeth Nauman, Eboni Price-Haywood, Cathy Glover, Patricia Dominick, Peggy Malone, Gang Hu, Lizheng Shi

**Affiliations:** 1 Department of Global Community Health and Behavioral Sciences, Tulane University School of Public Health and Tropical Medicine, New Orleans, LA 70112, USA; 2 Department of Global Health Management and Policy, Tulane University School of Public Health and Tropical Medicine, New Orleans, LA 70112, USA; mwharton@tulane.edu (M.K.W.); amonnett@tulane.edu (A.M.); lshi1@tulane.edu (L.S.); 3 Louisiana Public Health Institute, New Orleans, LA 70112, USA; bnauman@lphi.org; 4 Ochsner Health System Center for Applied Health Services Research, New Orleans, LA 70121, USA; eboni.pricehaywood@ochsner.org; 5 LEAD Study Steering Committee, New Orleans, LA 70112, USA; cglover780@aol.com (C.G.); pat.dominick@amequity.com (P.D.); pmalone@ochsner.org (P.M.); 6 Pennington Biomedical Research Center, Baton Rouge, LA 70808, USA; gang.hu@pbrc.edu

**Keywords:** health care quality, access, and evaluation, diabetes complications, disease management, health systems research, aged

## Abstract

The burden of illness related to diabetes and its complications is exceedingly high and growing globally. Systematic approaches to managing chronic care are needed to address the complex nature of the disease, taking into account health system structures. This study presents data collected from interviews with physicians, health system administrators, and other healthcare staff about chronic care management for elderly people with diabetes co-morbid with other chronic conditions in light of new programs intended to reduce barriers by incentivizing care encounters that take place through telephone and electronic communications (non-face-to-face care). Results indicate that health system personnel view non-face-to-face care as potentially providing value for patients and addressing systemic needs, yet challenging to implement in practice. Barriers and facilitators to this approach for managing diabetes and chronic care management for its complications are presented, with consideration to different types of health systems, and recommendations are provided for implementation.

## 1. Introduction

Approximately 30.3 million people in the US, an important proportion of the adult population, live with diabetes [1] and nearly 100 million live with multiple chronic diseases according to a 2017 study [2]. Complications from uncontrolled diabetes, including diabetic retinopathy, kidney failure, ulcers leading to foot and leg amputations, and other disabilities, contribute substantially to loss of quality of life for those suffering from diabetes and related chronic conditions [3]. Costs to the health care system as a result of diabetes are estimated at greater than $174 billion annually [1]. Complications are largely preventable with medication and behavioral interventions [4,5]. However, the disease remains prevalent in the US and globally, leaving health care services at the system and population levels challenged to deliver sustainable and effective interventions to decrease associated mortality and morbidity [6,7].

Chronic care management can improve the day to day experiences of people living with multiple chronic diseases like diabetes [2,8]. Chronic care management (CCM) includes clinical care coordination and support for managing self-care, such as personal coaching, group classes, reminders, and customized feedback [9]. Among people with diabetes, these approaches have been shown to improve risk factors such as lipid levels, blood pressure, and glycemic control, as well as outcomes including reduced hospitalization and avoided amputations [9,10,11,12,13,14,15,16]. CCM services can be delivered in person or remotely via information and communication technologies (phone, email, and electronic messaging) for non-face-to-face chronic care management (NFF CCM) [9,15]. Given that chronic care management is intended to improve both health service delivery and patient quality of life, research into facilitators of chronic care management has grown in recent years. This has resulted in the identification of several domains of interest: health information systems development [17,18,19], organized channels of communication [6,20,21], collaboration with specialists such as endocrinologists [22,23,24], relationship building with patients to improve engagement [6,25], involvement of community health workers and/or educators [20,26,27], telemedicine such as remote screenings [28,29,30], medication management [21,31], and establishment of clinic-community collaborations [6,18]. Potential barriers to implementing CCM programs include challenges addressing social needs of the patient that impact their self-management ability [6] and establishing provider buy-in for new processes and procedures [18].

Government initiatives have been introduced to incentivize health systems and physicians to deliver CCM services. One such initiative is a reimbursement policy established by the United States Centers for Medicare & Medicaid Services (CMS) in 2015 to incentivize health systems to provide NFF CCM for Medicare patients with two or more chronic conditions [32]. Several Current Procedural Terminology (CPT) billing codes provide CMS reimbursement for NFF CCM. These include $43 for at least 20 min of services that can be billed one time per month (CPT 99490), $90 for at least 60 min of services for more complex patients that can be billed one time per month (CPT 99487), and $47 for each additional 30 min of NFF CCM services in a month (CPT 99489) [32].

The current study is part of a larger natural experiment study to understand the impact of the CMS incentives on population health, diabetes, and chronic care management. Understanding barriers and facilitators to implementing new billing and care systems will complement quantification of health outcomes and changes that may result from NFF CCM programming. Previous work conducted in this setting sought to explore administrative and leadership support, staffing, and documentation for reimbursement as important contributors to successful CCM [33]. The current study seeks to build on that initial exploration to understand the perspectives of providers and health care system administrators and identify challenges and facilitators to the successful implementation of NFF CCM programs. These insights will result in actionable information for health systems and clinicians working in the field of diabetes and chronic disease care.

## 2. Methods

*Study design, setting and participants*: The Louisiana Experiment Assessing Diabetes (LEAD) project, of which this interpretive study [34] is a part, employs a natural experiment design using electronic health record data along with qualitative research with patients and health care providers to assess NFF CCM services per the Reach, Effectiveness, Adoption, Implementation, and Maintenance (RE-AIM) framework found at www.re-aim.org. Qualitative data collection was conducted from September 2017 to February 2018 utilizing semi-structured interviews to explore the experiences and views of clinicians and health system staff whose health system had or would soon be implementing NFF CCM program. Twenty participants were purposively selected within three major health care systems, and a group of four Federally Qualified Health Centers (FQHCs) in Southeastern Louisiana for maximum variation [35,36]. This study was approved by the Tulane University Institutional Review Board.

Purposive sampling was used in the identification of participants involved with the planning and delivery of NFF CCM services (physicians, health system leaders, nurses, and other clinic staff) and was facilitated by the researchers and partners on the LEAD Steering Committee. Chain referral sampling was also employed, and interviewees from the previous year of the study assisted in identification of other potential participants. Providers were then invited via email or phone to participate in interviews and give information about the study. If they agreed to be interviewed, participants were read an informed consent document, and their verbal or written consent was documented. No one else was present during the interviews other than the researchers and participants. A total of 43 providers and system leaders were contacted for recruitment, and of those, 20 participated in the study, rendering the response rate 46.5%.

*Semi structured interviews*: Interview guides for the semi-structured interviews were drafted by the authors and reviewed and revised by the LEAD Study Steering Committee, which includes physicians, academic researchers, patient partners, and representatives from payer organizations. Pretesting of the guide was completed to enhance validity. Data collection was piloted and refined prior to rollout. The interviewers were the first three authors of this study, and each researcher is affiliated with LEAD Study partner organizations with graduate-level training (one PhD level, three master’s level) in public health and experience in conducting focus groups and interviews in a variety of health-related settings. For the majority of interviews, no relationship was established between the interviewer and interviewee prior to the interview. Exceptions included when an interviewee was being interviewed again in the second year of the study by the same researcher. Interviewers read a scripted explanation of the LEAD Study, its purposes, and the interviewer’s role in the research to participants during the consent process at the beginning of the interview. Interviewers practiced self-reflexivity in acknowledging their biases and assumptions that may have impacted their interactions with participants during interviews and created field notes during and after each interview. The semi-structured interviews, conducted mainly by phone due to participant preference, lasted between 20 to 45 min, were recorded, and transcribed. Transcripts were deidentified and were reviewed by the researchers as they were received, but were not shared with participants. Comprehensive coverage of responses was discussed amongst researchers during the recruitment and preliminary review processes [35]. Participants received a nominal gift card in recognition of their contribution.

The transcripts were entered into NVivo® qualitative analysis software (QSR International Pty Ltd. Version 11, 2017, Melbourne, Australia). Analysis followed a descriptive, qualitative approach [37], and thematic analysis as illustrated by Braun and Clarke [38] was adapted to guide the coding and thematic development. Analysis was conducted by four experienced qualitative researchers. Open coding was used to suggest initial codes which were then expanded or revised before the development of categories and candidate themes. Initially, 59 codes were identified, discussed, and defined, which fell into 12 categories [35]. Candidate themes were considered and discussed in the context of reflexivity. Provider and health system leaders from the study steering committee, as well as patient partners, were included in debriefing sessions of the initial thematic findings and continued to provide feedback throughout the analysis. Peer debriefing, reflexive journaling in the form of memos, and feedback from study participants were all utilized to ensure trustworthiness of the data collection and analysis processes. Participant quotes are included in the results to exemplify study findings. The researchers found that the data were consistent with the findings and there was consensus amongst researchers on the clarity of the findings for both the major and minor themes identified [35].

## 3. Results

The 20 participants, whose characteristics are summarized in Table 1 below, were recruited from three different health systems (*n* = 10), non-profit primary care FQHCs (*n* = 8), and other clinics (*n* = 2). Of the sample, 5 participants (25%) had ever provided NFF CCM with the CMS CPT code(s) and 15 (75%) had never provided NFF CCM. The mean age of participants was 46.1 years (ranging from 32–68 years old), and 9 were male (45%) and 11 female (55%). By position within their organization, 12 participants (60%) were physicians, including primary care doctors, internists, and endocrinologists, 4 participants (20%) were nurses, all of whom were involved in CCM activities, and 4 participants (20%) were staff or administrators, including executive leadership, CCM and population health administrators, and billing specialists.

A range of themes and subthemes were identified relating to challenges involved in the successful development of NFF CCM programs, as well as facilitators to implementing the program. These are summarized in Table 2 and presented in more detail below.

One theme related to facilitators and benefits was the perception that NFF CCM adds value to the care provided to patients. The majority of participants acknowledged that education on chronic condition management was an important aspect of the spectrum of care patients with chronic conditions need, which could be met through NFF CCM. Quotations from participants related to this are illustrated below.

People have very interesting ideas as to what illnesses are when they don’t know much about them, especially when they know older folks that they’ve known and had them. It becomes a little bit just like a story. Someone shares their problems. That’s all they know. They really don’t get the differences between them, they don’t get the difference between being on insulin or not, they don’t understand necessarily the things that could go a long way towards improving the quality of their health.

(Primary care physician, male)

But the majority of the time we are constantly reinforcing the information. We’re explaining I would say out of 100 percent I would say we have maybe a 60 percent success rate and that’s across all demographics and ethnic groups. But we still struggle with trying to convince the rest of the 40 percent. And it’s an ongoing thing. It’s a constant.

(Nurse, female)

Moreover, providers described NFF CCM as a strategic use of resources in an already constrained environment and helpful with the allocation of staff time to best meet the needs of patients when used appropriately and optimally. The following quotes are illustrative:

It’s another way to kind of, you know, (to) use your resource in the best way. All of the population health stuff to me, the key essential thing is a recognition that you have a finite number of resources, an infinite amount of needs and how to match the resource with the most likely to benefit group.

(Primary care physician, male)

We try and do all avenues because the scale of it, like, the us to them is enormous…when you’ve got 40,000 diabetics and you’ve got 200 docs that doesn’t add up. I mean…unless we just all do diabetes only, you’re not going to get your hands around it.

(Health care administrator, male)

However, health care providers and health system leaders indicated that not all patients qualifying for the services would necessarily need them or benefit from them, and that some patients have support and resources while others do not. Participants described certain types of patients who might benefit the most from NFF CCM services:

Honestly the lower the health literacy I would think probably the more beneficial. I think in a lot of cases people aren’t necessarily willfully ignoring what [the] doctor’s saying. In a lot of cases people are just overwhelmed…especially diabetes takes up a lot of your life. Having diabetes is work.

(Health care administrator, female)

Staffing and communication processes were also identified as key to the efficiency of the program. Administrators described the roles of the different staff involved in NFF CCM service provision, such as nurses, coordinators, health coaches, and diabetes educators, and how their roles may vary. Consideration for differences in staff type and availability at various organizations such as teaching hospitals with residents providing care versus practices with permanent, long-term faculty and staff were also discussed.

In addition to the time needed by staff for direct patient contact, participants often cited time and effort for documentation required for reimbursement as a major barrier to initiating NFF CCM programs. Other areas mentioned were the required documentation of the patient encounters, and clarification of procedures, including care plan access, and communication with the care team.

It is just too burdensome, I don’t have time to sit down and review a silly ass care plan—and you can quote me on that—so we are not fully taking advantage of [CMS NFF CCM reimbursement].

(Physician, male)

Structural issues, such as centralized or decentralized programs, were also discussed by providers and health care systems. For instance, where a NFF CCM program is housed might impact “who gets to use it” or which patients get enrolled based on where they receive their care. Similarly, a health system may have more than one care management or preventive health program, which could cause confusion or duplication of effort.

One of the problems that we kind of run into and our patients run into is sometimes these initiatives are not necessarily coordinated either from within one specific disease it may not be coordinated or amongst various diseases. So, what I mean by that is, you know, you may have an initiative that’s done for diabetes, but pharmacy might be doing one thing, our innovation team may be doing one thing, our education team may be doing something else and then primary and endocrine. Then on top of that, most of these patients don’t just have diabetes, they have multiple other chronic diseases.

(Physician, male)

*Because in-clinic workflow actually does really have a large impact on what happens once the patient leaves your clinic. It truly does.*

(Health administrator, female)

Participants also identified size of an organization and scale of its NFF CCM program as an important indicator of its ability to be successful and sustainable. Health system administrators discussed issues of return on investment and cost effectiveness as a consideration of implementation strategies. The need to dedicate effort to documenting in order to bill for NFF CCM services emerged as an important issue. Differences between the resources available within larger health systems versus smaller organizations such as FQHCs arose, for example, access to disease registries for recruitment and information systems and technologies (such as appropriate Electronic Health Record systems) to document encounters for billing purposes.

So the things that we’re doing we’re not getting all of the credit because we’re not using the timer (within the electronic medical record). That’s been a big part of the challenge.

(Nurse, female)

Basically, in many cases we’ve been doing a lot of this, but never like, you know documenting, and now we’re really trying to…Okay. We tried this. We sent this. You know, all these little things we do to get care coordinated, but we’re really going to have like specific encounters (documented). So, actually any time we’re doing stuff we’re going to have to create this special encounter so that it’s tracking.

(Physician, female)

Also, scale and scope of work were addressed from the perspective of resource constraints and identification of optimal patients in health care settings where everyone who qualified was not able to be managed by small NFF CCM programs with limited staff. Many system leaders and administrators discussed cost effectiveness as “only hiring staff who can pay for themselves,” in the form of care coordinators who produce patient revenue at least equivalent to their salary in order to sustain the NFF CCM program with specially hired staff.

In terms of the care coordination I mean there are a lot of patients that have multiple comorbidities who, you know, really could use care coordination and I don’t see it used as much as probably needs to be and I’m not sure how to even make it happen personally.

(Physician, female)

The only obstacle that I see would be if the administration would see the utility of it and hiring competent, motivated personnel to do that.

(Physician, male)

Additionally, a theme emerged of NFF CCM not being financially sustainable, especially if there was low patient enrollment. Participants noted that this was leading some health systems to consider outsourcing their NFF CCM programs.

One health system’s response to resource constraints discussed in interviews was outsourcing NFF CCM services to one of many third-party vendors offering those services and using the CMS CPT codes by fee for service to the health care system. Some system leaders noted that using a third party can decrease the complications of NFF CCM provision as they only have one function, while being more cost effective with specialization in this function.

The idea of going with an outside company is they have expertise, they have infrastructure. For us to then build that infrastructure would take a large investment...when you change the scale, you have to go—you have to think in a completely different way. [The third party operators] are very realistic. Everything they talk about makes sense. I mean, like, they’re very pragmatic. And so I’m quite certain that if there is a code that they can use that’s more effective, I’m sure that they’ll use it.

(Physician, male)

However, some participants articulated hesitation about outsourcing NFF CCM services. The main concern that surfaced in the interviews was that a for-profit, third party entity may not be “…emotionally invested in getting our patients [on the phone or into an appointment]”.

Yeah, I think that, you know, the more the care coordinator is familiar with the patient and not so much— you know, if you contract it out to somebody else or an outside organization, I think it could get less personal…Because the patients may not know who they’re talking about, you know, whereas these people who are embedded in the clinic – their patients, actually, when they come to clinic can actually talk to them and meet with them face-to-face. I think there’s a lot of value in that.

(Physician, male)

But it looks a lot better when we have a member of our team reaching out to the patient than say having a nurse from Nebraska call.

(Health administrator, male)

‘Concierge’ appointment-setting service. So are they emotionally invested in getting Mrs. Johnson back? I don’t know. Maybe they are. But I feel much more comfortable with our people doing it.

(Health administrator, female)

Additionally, participants noted challenges with patient retention after six months because patient need for the NFF CCM services seemed to decrease. Providers and leaders also voiced challenges engaging hard-to-reach (and therefore very vulnerable) patients.

It was kind of consistent throughout the entire program for the first four to six months and then we found after about six months people kind of dropped off and then we spent more time calling…then coordinating care. There was great engagement up to six months and then patients, the feedback we got from them where they felt like medically they had been optimized, they’ve had education, we had gotten them a lot of social support and resources put in place. So after about six months they kind of fell off in being able to continue case management.

(Administrator, female)

So, maybe diabetics who have spent a certain amount of time with their primary care doctor and have maybe plateaued at their progress.

(Physician, male)

Lastly, some providers and system leaders identified important issues for improving diabetes outcomes that need to be addressed through referral to other types of care, and not only via CCM. Notably, these included the importance of including behavioral health services in care to address patients’ psycho-social issues that drive lifestyle problems and social isolation.

We don’t really like to call it non-compliance because a lot of that is there’s always some kind of underlying behavioral health component whether it’s anxiety, depression or other more extensive behavioral health issues that really creates the struggles.

(Nurse care coordinator, female)

Also, providers, leaders, and care coordinators noted that patient compliance with diet and exercise recommendations may necessitate a health coach, nutrition counselor, registered dietician, or diabetes educator, and these are beyond the scope of the care coordinators employed for NFF CCM programs.

Because what I find is we have quite a few patients, they really want to do the right thing. They ask the questions, the desire is there, it’s just the means, the resources to sometimes do, to make the healthy choices, those are the things that are lacking. And if we are aware of resources in the community that will help vouchers or what have you that might be available to them, that will help and I know they would appreciate it and would benefit from it and would take use of it.

(Nurse administrator, female)

## 4. Discussion

Non-face-to-face CCM services have been recognized by CMS as a critical component of primary care that contributes to better health and care for elderly individuals with diabetes and other chronic conditions. In order to ensure that this care may be provided to patients, policies and practices have been introduced to lower the barriers for provision of care. This study identified themes related to challenges and facilitators to implementing NFF CCM programs within health systems where older patients with diabetes and other chronic health conditions would benefit from such services.

The current study findings are unique in that they derive from health system participants in the South, where the majority of elderly beneficiaries in the first two years of the CMS reimbursement program reside, according to a Mathematica Policy Research Institute report recently published. The report detailed the diffusion and impact of the services being reimbursed for since 2015 [39]. Additionally, the current study indicates a movement towards finding solutions for challenges encountered in the implementation or planning of NFF CCM programs for elderly patients with diabetes, where health systems find the programs appealing but difficult to manage. Other recent studies of care systems for patients with diabetes and its complications have focused on: (1) improving patient engagement; (2) communication and information sharing between patient, provider, and care team; and (3) coordination of care across settings including community partners [6,20,24]. The present study identified some of these as being important, while highlighting other factors as well, such as value for patients and needs that extend beyond CCM.

Management of diabetes among older adults is complicated and includes issues related to cognitive and functional decline, and diabetes management in this population may have different aims than in younger populations. A study by Lewis and colleagues found that care management enhanced patient empowerment and addressed local barriers and individual needs with coordination of resources from a variety of settings [7], whereas the present results indicated that coordination of multiple programs addressing diabetes and its complications may hinder accessing the benefits of care management due to complex systems. Another qualitative study focusing on care coordination for insulin initiation in patients with diabetes explored different clinician perceptions of roles as they pertained to developing relationships with clear communication, trust, and respect [18]. Our results indicated that physicians and health system staff are often overwhelmed by the large volume of patients, but also concerned that patients feel they are receiving care from someone that they know and trust, as opposed to someone from another region of the country. Similar to the findings in this study related to having appropriate staffing, another study identified confidence in the competency of CCM team members as being essential to success [21]. The current study found that providers valued CCM as a key component of care that supports patient self-management of their chronic conditions. Sharing of electronic health data as a necessary facilitator of CCM was a consistent finding for this study and in the wider literature [17,18,23,24] on CCM. In contrast to this study which focused on NFF encounters in primary care, another qualitative study on care management focused the importance and the limitations of EHR in the coordination of care across practices [24].

Future directions for NFF CCM include examining the integration of telehealth into primary care practices for management of diabetes care [30]. One study found the integration of pharmacists for medication management as a valuable component of care management programming, which represents another opportunity for NFF CCM [31]. Also, the inclusion of other health care specialists important to the care of patients with diabetes, such as dentists for diabetic oral health education and optometrists for retinopathy screening [28,29], could contribute to improving care and outcomes. Finally, inclusion of community health workers in CCM has been shown to improve patient engagement and outcomes in the treatment and prevention of diabetes [27,28]. Utilizing community health workers may be a complementary approach to addressing patients’ complex and diverse social needs, which were evident in the perspectives of care coordinators, providers, and leaders in this study.

Diabetes and other chronic illnesses remain prevalent in the United States and contribute to disease-related morbidity and mortality that produce loss of quality adjusted life years [2,3] and cost billions of dollars to the health care delivery system [2]. The identification of effective and sustainable interventions to attenuate these negative consequences of diabetes and related conditions is essential [6,7]. With the proliferation of evidence-based interventions to promote diabetes and other chronic condition management comes a need to better understand and tailor interventions to individual organizations and patient populations in order to ensure success and sustainability.

Based on the study results, the need to develop a clear strategy to recruit elderly patients who would benefit most from the program is clear. Not all patients will benefit equally from this program. Some patients are better able to organize their care and health in such a way that the benefit would be minimal compared to others such as newly diagnosed patients and those with more complex needs or fewer resources. There may additionally be a six-month threshold or “plateau” after which patient benefit from NFF CCM diminishes with stabilization, and the time investment might be better spent on new or more complex patients. While it will be necessary to sustain NFF CCM programs from a cost effectiveness perspective, attention to the issue of patient engagement among the elderly and quality of care must be balanced against the incentive to outsource or contract a third-party vendor. Health systems must cautiously consider strategy in light of maintaining patient-centered care. The issue of outsourcing NFF CCM could be productively evaluated in future studies, as more experience is accumulated with outside vendors. Connection and coordination to other needed services remains an important consideration when implementing NFF CCM in this population where cognitive and functional decline must be factored into diabetes management. Health systems must ensure that linkage and coordination to support auxiliary health services are available—behavioral health and psycho-social services could be provided to ensure that patients and health systems do not rely on NFF CCM to meet such additional needs. Some complex patients will benefit from a health coach, nutrition counselor, registered dietician, or diabetes educator, and provision of those services through care coordinators could be the most important benefit of an NFF CCM program.

Strengths of the study included being conducted by an experienced qualitative research team, trained in using validated methodology for exploring experiences of participants, and use of the Consolidated Criteria for Reporting Qualitative Research (COREQ) checklist for reporting qualitative research. Limitations include the inability to generalize findings widely beyond this study site, as is common to qualitative inquiry. However, the similarity of findings across health systems suggests that some findings may be more widely generalizable. There is also the potential for reporting bias in the study, where participants may have reported certain experiences to researchers while not mentioning others.

## 5. Conclusions

In-depth exploration of these issues demonstrates the complexity of caring for elderly populations with diabetes and other chronic conditions. NFF CCM program development and implementation may be crucial to managing older patients, along with the corresponding reimbursement for time spent in managment. Yet the potential of improved clinical biomarkers (such as hemoglobin, blood pressure, and LDL cholesterol [9]) and quality of life must be balanced against challenges of providing services. Due to differences between varied health care systems, no uniform set of recommendations would be appropriate, however, several important areas of NFF CCM service recommendations may be drawn from this study. For operational environments planning to utilize NFF CCM, three recommendations are provided: (1) the need to ensure clear patient recruitment and retention strategies in advance of implementing a program; (2) cautious movement towards a sustainability strategy; and (3) attention to comprehensive services linked with CCM to meet patient needs.

## Figures and Tables

**Table 1:** Descriptive Information on Providers and Leaders Interviewed for the LEAD (Louisiana Experiment Assessing Diabetes) Study (*n* = 20), 2017–2018. NFF CCM = non-face-to-face chronic care management.

Descriptive Information on Providers and Leaders Interviewed for LEAD Study (*n* = 20), 2017–2018		
	Frequency or Mean	Percent of Total
**Health System**		
Ochsner Health System	4	20%
Tulane Medical Center	5	25%
University Medical Center New Orleans	1	5%
Federally Qualified Health Centers (FQHC)	8	40%
Other	2	10%
**Ever provided NFF CCM**		
Ever provided NFF CCM	5	25%
Never provided NFF CCM	15	75%
**Age**		
Years	46.1	
**Sex**		
Male	9	45%
Female	11	55%
**Position Type**		
Physicians	12	60%
Nurses	4	20%
Administrators	4	20%

**Table 2:** Themes related to barriers and facilitators identified by participants.

Barriers	Facilitators
Burden on staff and time commitment	Adds value to care that patients receive
Communication among staff and existence of other similar programs	Represents a strategic use of resources
Financial sustainability	Electronic health records and scale of large healthcare organizations
Patient needs that extend beyond CCM (e.g., dietetics and behavioral health)	Appropriate staffing and expertise in billing (e.g., a 3rd party vendor specialized in NFF CCM)
Selection and retention of patients	
